# Cytosolic ATP Relieves Voltage-Dependent Inactivation of T-Type Calcium Channels and Facilitates Excitability of Neurons in the Rat Central Medial Thalamus

**DOI:** 10.1523/ENEURO.0016-18.2018

**Published:** 2018-02-15

**Authors:** Tamara Timic Stamenic, Slobodan M. Todorovic

**Affiliations:** 1 Department of Anesthesiology, University of Colorado, Aurora, CO 80045; 2 Neuroscience Graduate Program, University of Colorado, Aurora, CO 80045

**Keywords:** ATP, calcium, low-voltage activated, thalamus

## Abstract

The central medial nucleus (CeM) is a part of the intralaminar thalamus, which is involved in the control of arousal and sensory processing. However, ionic conductances and mechanisms that regulate the activity of the CeM are not well studied. Here, we used *in vitro* electrophysiology in acute brain slices from adolescent rats to demonstrate that T-type calcium currents (T-currents) are prominent in the majority of the studied CeM neurons and are critical determinants of low-threshold calcium spikes (LTSs), which in turn regulate excitability of these neurons. Using an ATP-free internal solution decreased T-current density and induced a profound hyperpolarizing shift in steady-state inactivation curves while voltage-dependent activation kinetics were spared. Furthermore, selective pharmacological blockade of T-channels or use of an ATP-free solution reduced both tonic action potential (AP) frequency and rebound burst firing in CeM neurons. Our results indicate that T-channels are critical regulators of a thalamocortical circuit output and suggest that cytosolic ATP could be an endogenous regulatory mechanism in which T-channels may functionally gate sensory transmission and arousal *in vivo*.

## Significance Statement

Recent studies have revealed the important impact of the central medial nucleus (CeM) of thalamus on control of arousal, yet the key ion channel that regulates its excitability has not been well studied. Here, we used patch-clamp recordings from acute brain slices to demonstrate for the first time that T-channels play an important supportive role in regulation of excitability of rat CeM neurons. Additionally, we found that T-channels not only play a major role in shaping the output of the CeM but also that activity is strongly regulated by cytosolic ATP. Hence, ATP may be an endogenous regulatory mechanism in which T-channels can functionally gate sensory transmission and arousal *in vivo*.

## Introduction

Voltage-gated calcium channels (VGCCs) play an important role in neuronal excitability, synaptic plasticity and neurotransmitter release (Leresche and Lambert, 2017). Two major classes of VGCCs are recognized based on their electrophysiological properties: T-type calcium (low-voltage-activated, T-type) and high-voltage-activated (HVA) channels. T-channels require a smaller depolarization for opening, exhibit relatively fast voltage-dependent inactivation and at resting membrane potential there is a small fraction of channels available for opening to form so called “window” currents (Crunelli et al., 2005). Three subtypes of T-type channels have been identified with different expression, co-localization in the nervous system, and contributions to neuronal firing, Ca_V_3.1, Ca_V_3.2, and Ca_V_3.3 (Chemin et al., 2002; Snutch and Cain, 2010). Consequently, T-type channel dysfunction has been implicated in sleep disorders, pain, absence epilepsy, cognitive functions, Parkinson’s disease, neuropsychiatric disorders and control of consciousness (Huc et al., 2009; Miwa and Kondo, 2011; Cheong and Shin, 2013; Chen et al., 2014). T-type channels are essential for generation of low-threshold calcium spikes (LTSs) that are well characterized in recordings from a variety of brain regions. It has been shown that LTS in thalamocortical glutamatergic neurons in various thalamic nuclei are mediated largely by the Ca_V_3.1 isoform of T-type channels (for review, see Perez-Reyes, 2003).

The central medial nucleus (CeM) of thalamus is part of the “nonspecific” intralaminar complex, which is part of the limbic nuclei of thalamus that sends diffuse glutamatergic projections to the cortex (Vertes et al., 2015). Recent studies have revealed the important impact of this nucleus on control of arousal (for review, see Saalmann, 2014), one of which is the role of the thalamus in inducing and maintaining general anesthesia. It has been shown that during induction of general anesthesia and transition into sleep, the CeM acts as a key hub through which general anesthesia and natural sleep are initiated (Baker et al., 2014). Additionally, the CeM has been identified as the neuroanatomic site mediating the arousal response; microinfusion into the CeM with nicotine and/or an antibody designed specifically to block K_V_1.2 voltage-gated potassium channels, or an inhibitor of shaker-related potassium channels (ShK toxin) can awaken anesthetized animals in the presence of an anesthetic agent (Alkire et al., 2007; Alkire et al., 2009; Lioudyno et al., 2013). Furthermore, the CeM is additionally implicated in nociception, regulation of seizures, and fear conditioning (Miller and Ferrendelli, 1990; Van Der Werf et al., 2002; Vertes et al., 2015). However, despite its important roles in the regulation of the state of arousal, sezures and nociception, the key ion channel that regulates excitability of the CeM has not been well studied.

Neuronal T-channels regulate sleep and wakefulness (Crunelli et al., 2014) and are inhibited by relevant concentrations of some general anesthetics (Todorovic et al., 2000; Joksovic et al., 2005; Orestes and Todorovic, 2010; Eckle et al., 2012). However, the possible role of T-channels in neuronal excitability of CeM neurons and regulation of their function with endogenous substances is not currently known. Here, we used patch-clamp recordings from acute brain slices to demonstrate for the first time that T-channels play an important supportive role in regulation of excitability of rat CeM neurons. Additionally, we found that T-channels not only play a major role in shaping the output of the CeM, but also that activity is strongly regulated by intracellular ATP. Hence, ATP could be an endogenous regulatory mechanism in which T-channels can functionally gate sensory transmission and arousal *in vivo*.

## Materials and Methods

### Animals

Experimental procedures with animals were performed according to the guidelines approved by University of Colorado Anschutz Medical Campus. Treatments of rats adhered to guidelines set forth in the National Institutes of Health Guide for the Care and Use of Laboratory Animals. All efforts were made to minimize animal suffering and to use only the number of animals necessary to produce reliable scientific data. Male and female Sprague Dawley rats (P = postnatal day, P21–P35) were obtained from Envigo (Indianapolis, IN, USA). All animals were maintained on a 12/12 h light/dark cycle with food and water *ad libitum*.

### *In vitro* brain slice preparation

Rats of either sex were anesthetized briefly with isoflurane and decapitated, and their brains were removed rapidly and placed in a cold (4°C) oxygenated (95 vol% O_2_ and 5 vol% CO_2_) solution. Live 250- to 300-μm-thick coronal brain slices were sectioned at 4°C in the same cold solution: 260 mM sucrose, 10 mM D-glucose, 26 mM NaHCO_3_, 1.25 mM NaH_2_PO_4_, 3 mM KCl, 2 mM CaCl_2_, and 2 mM MgCl_2_, using a vibrating micro slicer (Laica VT 1200S). Brain slices were immediately incubated for 30 min in the following solution: 124 mM NaCl, 10 mM D-glucose, 26 mM NaHCO_3_, 1.25 mM NaH_2_PO_4_, 4 mM KCl, 2 mM CaCl_2_, and 2 mM MgCl_2_ at 37°C before use in electrophysiology experiments, which were done at room temperature. During incubation, slices were constantly perfused with a gas mixture of 95 vol% O_2_ and 5 vol% CO_2_.

### Electrophysiology experiments

The external solution for voltage and current-clamp electrophysiology experiments consisted of the following: 125 mM NaCl, 25 mM D-glucose, 25 mM NaHCO_3_, 1.25 mM NaH_2_PO_4_, 2.5 mM KCl, 1 mM MgCl_2_, and 2 mM CaCl_2_. For voltage-clamp experiments, tetrodotoxin (TTX; 1 μM) was added to the extracellular medium as a voltage-dependent sodium current blocker, while current-clamp experiments contained the synaptic blockers picrotoxin (20 µM), d-AP5 (50 µM), and 2,3-dihydroxy-6-nitro-7-sulfamoyl-benzo[f]quinoxaline-2,3-dione (NBQX; 5 µM) in the extracellular medium. For current-clamp experiments, the internal solution with ATP consisted of the following: 130 mM potassium-D-gluconate, 5 mM ethylene-glicol-bis(*β*-aminoethylether)*N,N,N’,N’*-tetraacetic acid (EGTA), 4 mM NaCl, 0.5 mM CaCl_2_, 10 mM HEPES, 2 mM Mg-ATP, and 0.5 mM Tris-GTP (pH 7.2); 2 mM Mg-ATP and 0.5 mM Tris-GTP were omitted in the ATP-free solution (Table 1).

**Table 1.** The molarity and components of TMA-based and Cs-based internal solutions used for voltage-clamp recordings and internal solutions used for current-clamp recordings

TMA ATP-free		TMA with ATP		Cs ATP-free		Cs with ATP		Internal for current clamp ATP-free		Internal for current clamp	
Molarity	Component	Molarity	Component	Molarity	Component	Molarity	Component	Molarity	Component	Molarity	Component
135 mM	TMA-OH × 5 H_2_O	135 mM	TMA-OH x 5 H_2_O	110 mM	Cs methane sulfonate	110 mM	Cs methane sulfonate	130 mM	K-D-gluconate	130 mM	K-D-gluconate
40 mM	HEPES	40 mM	HEPES	10 mM	HEPES	10 mM	HEPES	5 mM	EGTA	5 mM	EGTA
10 mM	EGTA	10 mM	EGTA	9 mM	EGTA	9 mM	EGTA	4 mM	NaCl	4 mM	NaCl
2 mM	MgCl_2_ × 6 H_2_O	14 mM	Phosphocreatine di-tris			14 mM	Phosphocreatine di-tris	0.5 mM	CaCl_2_ x 2 H_2_O	0.5 mM	CaCl_2_ × 2 H_2_O
		5 mM	Mg-ATP			5 mM	Mg-ATP	10 mM	HEPES	10 mM	HEPES
		0.3 mM	Tris-GTP			0.3 mM	Tris-GTP			2 mM	Mg-ATP
										0.5 mM	Tris-GTP

Abbreviations for different components of internal solutions are defined in Materials and Methods.

To facilitate HVA current run down we used a fluoride (F-)-based intracellular solution with tetramethylammonium-OH (TMA); the TMA ATP-free internal solution for voltage-clamp experiments consisted of the following: 135 mM TMA, 10 mM EGTA, 2 mM MgCl_2_, and 40 mM HEPES; titrated to pH 7.15–7.20 with hydrofluoric acid (HF). The TMA with ATP solution consisted of the following: 135 mM TMA, 10 mM EGTA, 40 mM HEPES, 14 mM phosphocreatine, 5 mM Mg-ATP, and 0.3 mM Tris-GTP; titrated to pH 7.15–7.20 with HF (Table 1; Todorovic and Lingle, 1998).

The internal solution solution for voltage-clamp experiments with Cesium (Cs) containing ATP consisted of the following: 110 mM Cs-methanesulfonate, 14 mM phosphocreatine, 10 mM HEPES, 9 mM EGTA, 5 mM Mg-ATP, and 0.3 mM Tris-GTP; pH adjusted to 7.15–7.20 with CsOH (standard osmolarity: 300 mOsm; Todorovic et al., 2000). The Cs ATP-free internal solution consisted of the following: 110 mM Cs-methane sulfonate, 10 mM HEPES, and 9 mM EGTA; pH adjusted to 7.15–7.20 with CsOH (Table 1). Because all our ATP-containing solutions had both ATP and GTP components, in some experiments we recorded with a Cs-based internal solution that contained only 5 mM Mg-ATP but not 0.3 mM Tris-GTP.

Whole-cell recordings were performed in CeM neurons visualized under Zeiss optics (Zeiss AXIO Examiner D1, 40× objective). Glass microelectrodes (Sutter Instruments, borosilicate glass with filament OD 1.2 mm) were pulled using a Sutter Instruments P-1000 model and fabricated to maintain an initial resistance of 3–6 MΩ. Neuronal membrane responses were recorded using a Multiclamp 700 B amplifier (Molecular Devices). Voltage current commands and digitization of the resulting voltages and currents were performed with Clampex 8.3 software (Molecular Devices) running on a PC-compatible computer. Resulting current and voltage traces were analyzed using Clampfit 10.5 (Molecular Devices). Statistical and graphical analyses were performed using GraphPad Prism 7.0 software (GraphPad Software) or Origin 7.0 (OriginLab). Results typically are presented as mean ± SEM unless stated otherwise.

### Voltage-clamp experiments

T-channel activation was measured by stepping the membrane potential from an initial holding potential (V_h_) of –90 mV to test potentials (V_t_) from –80 to –40 mV in 2.5-mV increments over a period of 320 ms. Current−voltage (I–V) curves were generated, and peak current amplitudes and inactivation properties of current waveforms were established and compared between internal solutions with ATP and without ATP. We calculated current densities by measuring average peak current divided by the capacitance of the neuron. Steady-state inactivation curves were generated by using a standard double-pulse protocol with 3.6-s-long prepulses to variable voltages (from −120 to −60 mV in 5-mV increments) and test potentials to −50 mV. The voltage dependencies of activation and steady-state inactivation were described with single Boltzmann distributions of the following forms:

Activation:G(V)=Gmax/(1+exp[−(V−V50)/k])

Inactivation:I(V)=Imax/(1+exp[−(V−V50)/k])

In these forms, I_max_ is the maximal amplitude of current, G_max_ is the maximal conductance (calculated by dividing current amplitude by estimated reversal potential), V_50_ is the voltage at which half of the current is activated or inactivated, and *k* represents the voltage dependence (slope) of the distribution.

To assess percentage of recovery from inactivation we used a paired-pulse protocol in which a 1-minute step to –50 mV was used to inactivate all of the T-type calcium currents (T-currents). After a variable recovery interval (from 2 to 10,000 ms) at either –90 or –120 mV, a second step to –50 mV was used to determine the amount of T-current that had recovered from the inactivation during the recovery protocol. Additionally, in experiments with the pharmacological blocker 3,5-dichloro-N-[1-(2,2-dimethyl-tetrahydro-pyran-4-ylmethyl)-4-fluoro-piperidin-4-ylmethyl]-benzamide (TTA-P2), a T-current was elicited by stepping to –50 mV from a holding potential of –90 mV every 20 s five times.

### Current-clamp experiments

Both tonic and burst-firing properties of CeM neurons were characterized by using multistep protocols. To investigate tonic firing patterns in CeM cells, we injected a depolarizing current pulse through the recording pipette of 400-ms duration in 25-pA incremental steps starting from 50 pA. To investigate burst-firing patterns, the neurons were injected with hyperpolarizing currents in 25-pA intervals stepping from 0 to −200 pA. Subsequent resting membrane potentials (RMP), tonic action potential (AP) frequencies, rebound APs, low-threshold spike (LTS) amplitude, latency to LTS, and input resistances were determined. Resting membrane potential was measured at the beginning of each recording and was not corrected for the liquid junction potential.

### Drugs

TTX and TTA-P2 were purchased from Alomone Lab. All other compounds were purchased from Sigma Chemical. Pan-selective T-type calcium channel blocker TTA-P2 was prepared as a 5 mM stock solution in dimethylsulfoxide (DMSO); aliquots were stored at –20°C and diluted for use at a final concentration of 5 μΜ, which was delivered with a gravity-driven perfusion system.

### Data analysis

In every *in vitro* experiment, we attempted to obtain as many neurons as possible from each animal to minimize the number of animals used. Statistical analysis was performed using one-way or two-way repeated measure (RM) ANOVA (in TTA-P2 experiments both factors were repeated), as well as Student unpaired and paired two-tailed *t* test where appropriate. Where interaction between factors after two-way RM ANOVA was significant, Sidak’s *post hoc* or uncorrected Fisher LSD comparisons were used. Tukey’s *post hoc* test was used following one-way ANOVA. Significance was accepted with *p* < 0.05. Statistical and graphical analysis was performed using GraphPad Prism 7.00 software (GraphPad Software) and Origin 7.0 (OriginLab).

## Results

### Biophysical properties of T-currents

**Figure 1.**
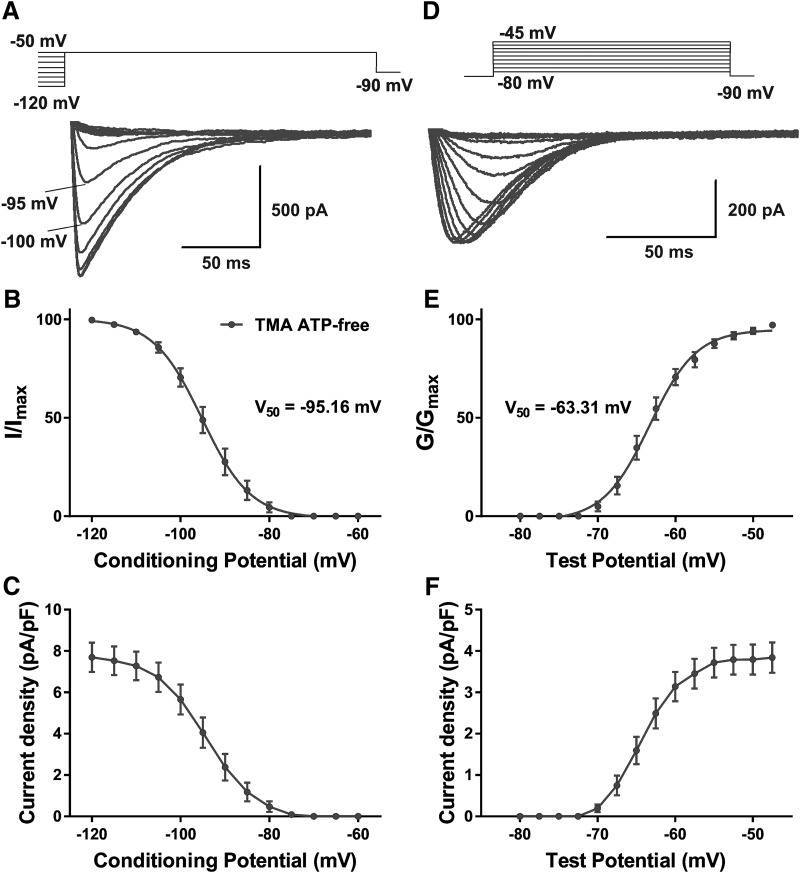
Biophysical properties of T-currents in the rat CeM. ***A***, T-current traces from representative CeM neurons generated using a double-pulse protocol with 3.6-s-long prepulses to variable voltages (from –120 to –50 mV in 5-mV increments) and a test potential (V_t_) of –50 mV recorded with TMA ATP-free internal solution. ***B***, The average steady-state inactivation (I/I_max_) curve; the V_50_ value is noted on the graph. ***C***, Average current density, as calculated from the steady-state inactivation protocol. ***D***, T-current I–V traces from representative CeM neurons in the voltage range for V_t_ of –80 to –45 mV from an initial holding potential (V_h_) of –90 mV in 2.5-mV increments recorded with TMA ATP-free internal solution. ***E***, The average voltage dependence of a steady-state activation (G/G_max_) curve with V_50_ value noted on the graph. ***F***, Average current density from multiple I–V curves.

To examine the biophysical properties of well-isolated T-channels in the CeM, we used an internal solution with TMA and HF (TMA ATP-free solution). This allowed us to investigate T-current properties in virtual isolation from other types of VGCCs (Todorovic and Lingle, 1998). Essentially, all of the CeM neurons in our study displayed inward currents with typical properties of T-currents, as has been previously described for other thalamic regions; e.g., the ventro-basal (VB) nucleus (Eckle et al., 2012). Representative traces from voltage-dependent inactivation of T-currents of CeM neurons over a wide range of prepulse potentials are presented in Figure 1*A*. For experimental conditions using a TMA ATP-free internal solution, the average V_50_ value for steady-state inactivation was –95.16 ± 0.62 mV (17 cells, nine animals) with a slope factor of 5.49 ± 0.57 mV (Fig. 1*B*; Table 2) and a corresponding average maximal current density of 7.7 pA/pF (Fig. 1*C*). A representative trace from a current−voltage (I–V) experiment showing properties of voltage-dependent activation of T-currents is presented in Figure 1*D*. The average V_50_ value for T-current activation was –63.31 ± 0.31 mV (17 cells, nine animals) with respective slope factors of 3.00 ± 0.29 (Fig. 1*E*; Table 2). The maximal current density for current activation (Fig. 1*F*) was 3.8 pA/pF, which was lower than the value obtained for steady-state inactivation (Fig. 1*C*). Previous molecular studies using *in situ* hybridization have shown that the main isoform of T-channels in the CeM is Ca_V_3.1 (Talley et al., 1999). Indeed, a relatively fast time constant of T-current inactivation at the peak current activation of V_t_ = –47.5 mV (30.74 ± 1.21 ms, *n* = 17 neurons, nine animals) in our current–voltage relationships strongly suggests Ca_V_3.1 is the dominant expression in the rat CeM nucleus.

**Table 2.** Average V__50__ (mean ± SEM) values for steady-state activation and inactivation with different internal solutions in CeM neurons

	TMA ATP-free	TMA with ATP	Cs ATP-free	Cs with ATP	Cs with ATP GTP-free
V_50_ activation (number of cells)	–63.31 ± 0.31 (17)	–63.25 ± 0.42 (18)	–63.72 ± 0.40 (18)	–63.16 ± 0.39 (21)	–63.88 ± 0.64 (14)
V_50_ inactivation (number of cells)	–95.16 ± 0.62 (17)	–89.86 ± 0.41 (22)	–81.56 ± 1.11 (15)	–73.70 ± 0.64 (21)	–75.15 ± 0.58 (15)
Unpaired *t* test V_50_ inactivation	*t*_(37)_ = 7.36, *p* < 0.001*		*t*_(34)_ = 6.53, *p* < 0.001* t_(34)_ = 1.61, *p* = 0.117^ns^		
	*TMA ATP-free vs TMA with ATP		*Cs ATP-free vs Cs with ATP ^ns^Cs with ATP GTP-free vs Cs with ATP		

Note that inclusion of ATP shifted V_50_ for inactivation toward more depolarized potentials in both Cs-based and TMA-based solutions. In contrast, V_50_ for activation was minimaly affected in the same cells.

### ATP regulates current density and relieves voltage-dependent inactivation of T-channels

**Figure 2.**
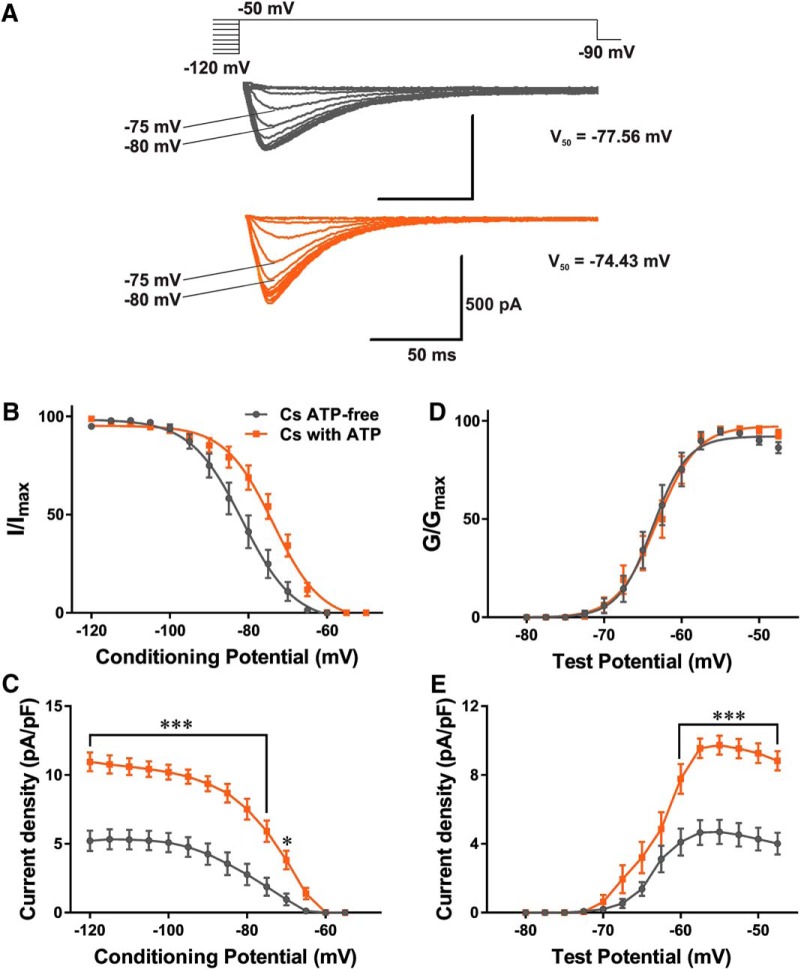
ATP regulates current density and relieves voltage-dependent inactivation of T-type calcium channels. ***A***, T-current traces from a representative CeM neuron with respective V_50_ values generated using a double-pulse protocol with 3.6-s-long prepulses for variable voltages (from −120 to −50 mV in 5-mV increments) and a test potential (V_t_) of −50 mV; gray traces recorded with a Cs ATP-free internal solution; and orange traces recorded with an internal solution of Cs with ATP. ***B***, Normalized average steady-state inactivation (I/I_max_) curve, depolarizing shift of 7.86 mV in a cohort of neurons with an internal solution of Cs with ATP was statistically significant from a cohort with Cs ATP-free internal solution (unpaired two-tailed *t* test, *t*_(34)_ = 6.53, *p* < 0.001). ***C***, Average current density, as calculated from the steady-state inactivation protocol with Cs-based internal solutions. Addition of ATP significantly increased current density by approximately two-fold [two-way RM ANOVA: interaction (*F*_(13,442)_ = 12.91, *p* < 0.001), voltage (*F*_(13,442)_ = 122.00, *p* < 0.001), and ATP (*F*_(1,34)_ = 33.71, *p* < 0.001, Sidak’s *post hoc* presented on graph)]. ***D***, The average normalized voltage dependence of steady-state activation (G/G_max_) in internal solution with ATP (orange line) and without ATP (black line) conditions. Note that the two curves overlap. ***E***, Average current density from multiple I–V curves, addition of ATP increases T-current density [two-way RM ANOVA: interaction (*F*_(13,481)_ = 9.68, *p* < 0.001), voltage (*F*_(13,481)_ = 79.44, *p* < 0.001), and ATP (*F*_(1,37)_ = 32.76, *p* < 0.001, Sidak’s *post hoc* presented on graph)]; *p < 0.05, ***p < 0.001.

We next investigated the effects of Cs-based internal solutions on T-channel kinetics in CeM. Interestingly, using different internal solutions containing ATP during T-current recordings in the CeM nucleus potentiated T-currents and altered inactivation kinetics. Families of representative traces from recordings designed to study properties of voltage-dependent inactivation with Cs-based internal solutions are presented in Figure 2*A*, gray traces, Cs ATP-free; orange traces, Cs with ATP. Figure 2*B* shows plots for the average steady-state inactivation curves and average V_50_ values –81.56 ± 1.11 mV (gray curve, Cs ATP-free; 15 cells, six animals) and –73.70 ± 0.64 mV (orange curve, Cs with ATP; 21 cells, seven animals). Slope factors for internal solutions without and with ATP were similar (6.64 ± 1.00 and 6.16 ± 0.57, respectively). The shift in steady-state inactivation of 7.86 mV in the cohort of neurons recorded with an ATP-containing internal solution was statistically significant compared with the cohort in an ATP-free solution (unpaired two-tailed *t* test, *t*_(34)_ = 6.53, *p* < 0.001). Correspondingly, addition of ATP in the Cs-based internal solution significantly increased current density by 45–50% over the wide range of conditioning potentials (Fig. 2*C*). In contrast to the shift observed in steady-state inactivation curves, there was very little change in voltage-dependence of steady-state activation of CeM neurons calculated from the current−voltage relationships. For example, the average V_50_ values derived from T-current steady-state activation were –63.72 ± 0.40 mV (Cs ATP-free, 18 cells, six animals) and –63.16 ± 0.39 mV (Cs with ATP, 21 cells, seven animals) with respective slope factors of 2.24 ± 0.36 and 2.59 ± 0.35 (Fig. 2*D*). Although there was no significant difference in voltage-dependent activation of T-currents between the two experimental conditions, addition of ATP did significantly increase maximal current density by approximately two-fold (Fig. 2*E*). In contrast, time constant of T-current inactivation at the V_t_ = –47.5 mV in our current−voltage relationships in control conditions (Cs with ATP, 37.72 ± 2.03 ms) was not significantly affected by the absence of ATP in internal solution (Cs ATP-free, 37.77 ± 1.96 ms, *p* > 0.05).

The average V_50_ values from T-current steady-state activation and inactivation with all TMA and Cs-based solutions are presented in Table 2. Similar to the findings with Cs-based internal solutions, experiments with TMA-based internal solutions confirmed the selective effect of ATP on steady-state inactivation kinetics. The average V_50_ value for steady-state inactivation was –95.16 ± 0.62 mV (TMA ATP-free, 17 cells, nine animals) and –89.86 ± 0.41 mV (TMA with ATP, 22 cells, four animals) with a slope factor of 5.49 ± 0.57 (TMA ATP-free) and 5.04 ± 0.38 (TMA with ATP). The 5.6 mV shift in steady-state inactivation was statistically significant (unpaired two-tailed *t* test, *t*_(37)_ = 7.36, *p* < 0.001). On the other hand, there was no difference between the average V_50_ values for steady-state inactivation kinetics recorded with Cs with ATP, GTP solution (–73.70 ± 0.64, 21 cells, seven animals) and Cs with ATP but GTP-free solution (–75.15 ± 0.58, 15 cells, three animals). Importantly, the voltage-dependent activation kinetics were virtually identical in all recording conditions as indicated by the V_50_ values, which remained stable at close to –63 mV.

### ATP modulates recovery from inactivation of isolated T-currents

**Figure 3.**
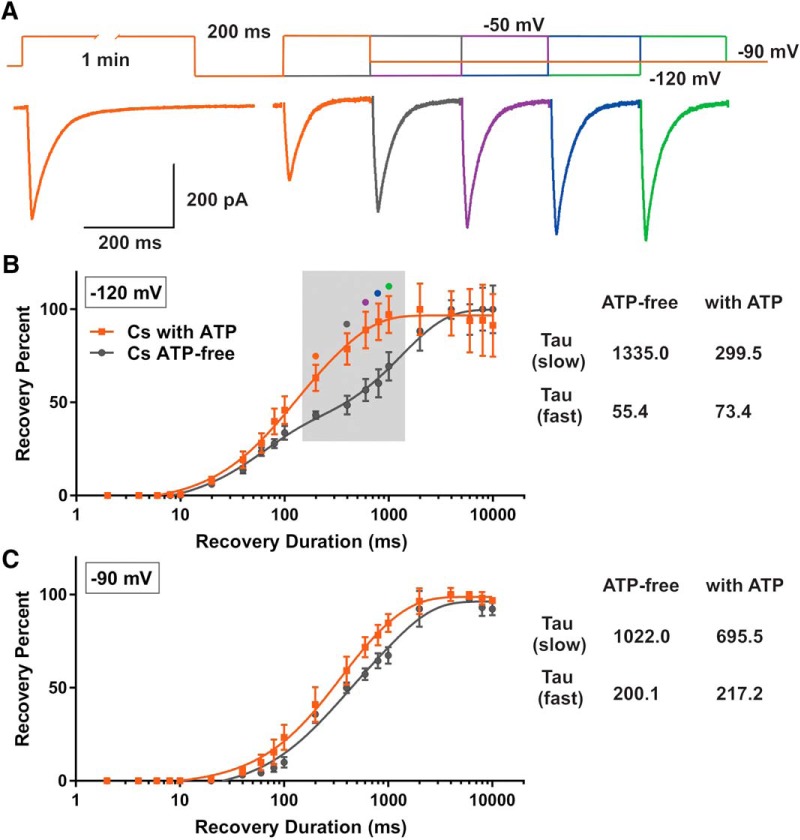
ATP modulates recovery from inactivation of isolated T-currents. ***A***, T-current traces from a representative CeM neuron generated using a paired-pulse protocol (top). Original traces show recovery from inactivation conditions with ATP at –120 mV, which lasted from 200 to 1000 ms (bottom); orange trace, 200 ms; gray trace, 400 ms; violet trace, 600 ms; blue trace, 800 ms; and green trace, 1000 ms. ***B***, Recovery time courses with an ATP internal solution (orange) and an ATP-free internal solution (gray) fitted with a double exponential function at –120 mV with respective time constants tau (τ). Gray box indicates significantly different data points in two recording conditions. ***C***, Recovery time courses with an ATP internal solution (orange) and ATP-free solution (gray) fitted with a double exponential function at –90 mV with respective time constants (τ). Note that recovery from inactivation was slower with the ATP-free internal solution solution at –120 mV than at –90 mV.

It is well established that T-type calcium channels must go through hyperpolarized membrane potentials to recover from inactivation. As a result of our voltage-clamp experiments, which demonstrated ATP potentiated T-currents and changed steady-state inactivation kinetics, we next examined ATP effects on recovery from inactivation of T-currents. We reasoned that ATP might influence excitability of CeM neurons by changing recovery from inactivation and availability of T-channels at different potentials. Because the duration of the preceding inactivation step determines the recovery rates from the inactivation state of the T-channels, we held CeM neurons at –50 mV for 1 min before increasing the length of recovery intervals at –90 and –120 mV (Joksovic and Todorovic, 2010). Recovery time courses with Cs-based internal solutions were fitted with a double exponential function yielding slow and fast time constants tau (τ). Representative traces showing T-current recovery from inactivation with a Cs-ATP containing internal solution at –120 mV (recovery interval from 200 to 10,000 ms) are presented at Figure 3*A*, gray trace, Cs ATP-free; orange trace, Cs with ATP; four to six cells, four animals. Figure 3*B* shows that recovery from inactivation with internal solutions that contain ATP (orange line and data points) was significantly faster at –120 mV by ∼4-fold, when compared to recovery in an ATP-free internal solution (black line and data points). A similar effect was detected when recovery from inactivation was recorded at –90 mV (Fig. 3*C*), although recovery time was slower than at –120 mV. The time constants presented in Figure 3*B*,*C* summarize the effects of ATP on T-currents with regards to recovery from inactivation at membrane potentials of –120 and –90 mV, respectively. Note that the absence of ATP had a greater effect on slow than fast time constants at both –120 and at –90 mV. As expected, the baseline fast time constant at –90 mV was noticeably slower in comparison to baseline at –120 mV.

### Inhibition of T-currents by TTA-P2

**Figure 4.**
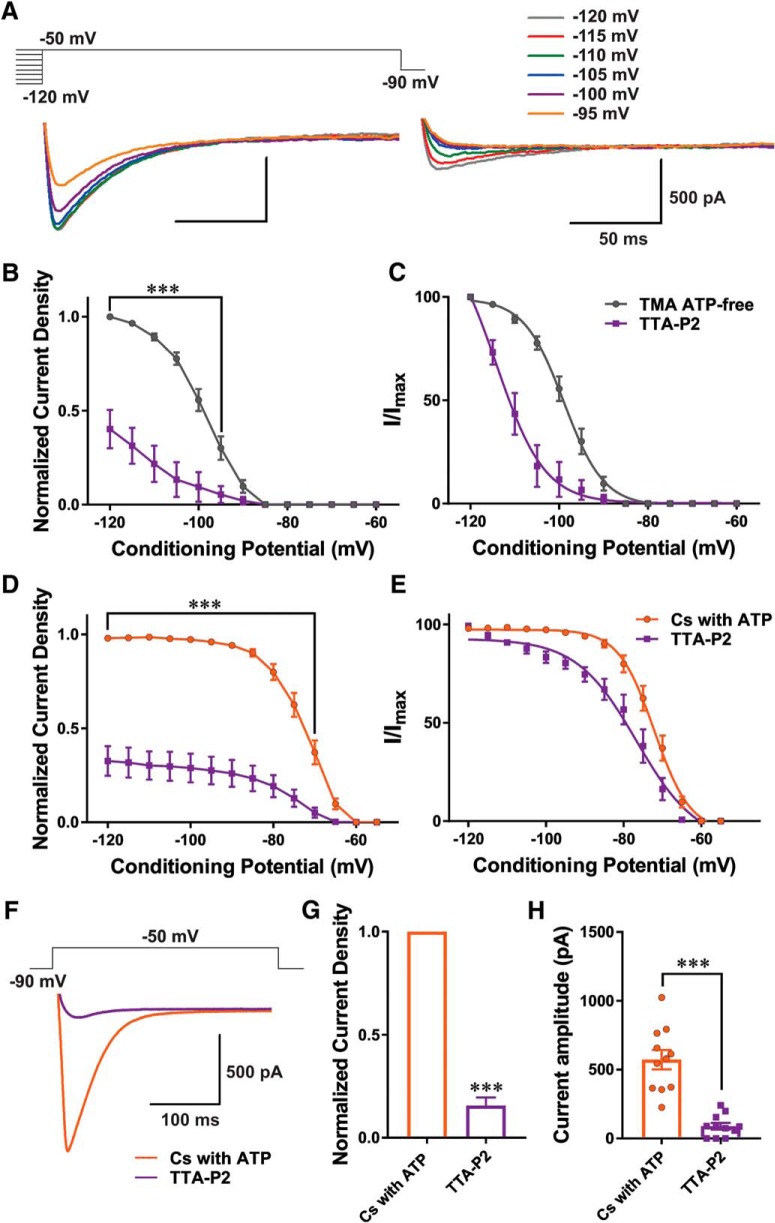
Mechanisms of T-current inhibition by TTA-P2. ***A***, Traces of inward calcium current in a representative CeM neuron in control conditions recorded with TMA ATP-free internal solution using a double-pulse protocol with 3.6-s-long prepulses to variable voltages; left panel, from –120 to –95 mV in 5-mV increments (control); right panel, traces from the same cell using the identical voltage-protocol during an apparent steady-state inhibition of T-current in the presence of 5 μM TTA-P2. ***B***, Average normalized current density, as calculated from the steady-state inactivation protocol. The presence of TTA-P2 (violet line and data points) decreased current density by 60–65% in comparison to the control conditions (gray line and data points). Data were analyzed with two-way RM ANOVA [interaction (*F*_(12,96)_ = 36.09, *p* < 0.001), voltage (*F*_(12,96)_ = 98.41, *p* < 0.001), and TTA-P2 (*F*_(1,8)_ = 41.34, *p* < 0.001, Sidak’s *post hoc* presented on figure)]. ***C***, Average normalized steady-state inactivation (I/I_max_) curves in control conditions and after application of TTA-P2 in the same cells. TTA-P2 induced a large hyperpolarizing shift in V_50_ of 15.26 mV (paired two-tailed *t* test, *t*_(8)_ = 6.82, *p* < 0.001). ***D***, Average normalized current density, as calculated from the steady-state inactivation protocol recorded with Cs with ATP internal solution before (orange line and data points) and after application of TTA-P2 (violet line and data points). TTA-P2 reduced current density by 65% (two-way RM ANOVA: interaction (*F*_(13,104)_ = 55.40, *p* < 0.001), voltage (*F*_(13,104)_ = 114.40, *p* < 0.001), and TTA-P2 (*F*_(1,8)_ = 73.38, *p* < 0.001, Sidak’s *post hoc* presented on figure)]. ***E***, Average steady-state inactivation (I/I_max_) curves in control conditions and after TTA-P2 recorded with Cs-based internal solution. TTA-P2 induced a significant hyperpolarizing shift in V_50_ of 4.55 mV (paired two-tailed *t* test, *t*_(8)_ = 2.77, *p* = 0.024). ***F***, Averaged representative traces recorded with Cs with ATP internal solution under control conditions (orange trace) and after application of TTA-P2 (violet trace) using a protocol depicted on the top of traces (V_t_ = –50 mV, V_h_ = –90 mV). ***G***, Averaged normalized current density show reduction in current density by 84% after application of TTA-P2 (paired two-tailed *t* test, *t*_(10)_ = 21.51, *p* < 0.001). ***H***, Averaged current amplitude under control condition was 572.75 ± 70.66 pA and after application of TTA-P2 it was 89.30 ± 24.21 pA (paired two-tailed *t* test, *t*_(10)_ = 7.01, *p* < 0.001); ***p < 0.001.

To exclude off-target effects, such as Ca_V_2.3 R-type of VGCCs (Choe et al., 2011), we used TTA-P2 as a pan-selective antagonist of T-channels at low micromolar concentrations (e.g., 5 μM). Representative current traces of a steady-state inactivation protocol (from –120 to –95 mV) recorded with TMA ATP-free solution are presented in Figure 4*A*; left panel shows traces of inward calcium current of a CeM neuron under control conditions; right panel shows traces from the same cell in the presence of 5 μM TTA-P2. Perfusion with TTA-P2 resulted in a significant reduction of normalized current density by 60–65% compared with control conditions (Fig. 4*B*). Addition of TTA-P2 to the internal solution stabilized inactive states of the channels as evidenced by a large hyperpolarizing shift in inactivation V_50_ values of ∼15 mV (Fig. 4*C*). The average V_50_ value for steady-state inactivation was –98.94 ± 0.44 mV with slope factor of 4.50 ± 0.39 in control conditions (Fig. 4*B*; pre-drug, nine cells, six animals). After TTA-P2 perfusion of the same cells, the V_50_ value was –114.20 ± 2.83 mV with slope factor of 5.48 ± 1.28 (Fig. 4*C*). TTA-P2 also profoundly reduced current density recorded with an internal solution of Cs with ATP during steady-state inactivation protocols as depiced on Figure 4*D*. Figure 4*E* depicts normalized voltage-dependent inactivation recorded with Cs-based internal solution. We found an average V_50_ value for steady-state inactivation of –72.10 ± 0.51 mV and slope factor of 4.64 ± 0.43 in control conditions (nine cells, seven animals); after TTA-P2 perfusion, the V_50_ value in the same cells was –76.65 ± 1.39 mV with slope factor of 7.76 ± 1.17. This hyperpolarizing shift of 4.55 mV was statistically significant (paired two-tailed *t* test, *t*_(8)_ = 2.77, *p* = 0.024). Additionally, the slope factor was significantly different after TTA-P2 perfusion (paired two-tailed *t* test, *t*_(8)_ = 2.33, *p* = 0.048). In contrast, little difference was seen in voltage-dependence of activation of T-currents in CeM neurons in the identical recording conditions after addition of TTA-P2. The average V_50_ of current activation value for control was –57.54 ± 0.45 with slope factor of 2.35 ± 0.39 (Cs with ATP, eight cells) and after application of TTA-P2 the V_50_ value was –57.20 ± 0.66 with slope factor of 2.71 ± 0.56 (data not shown). Representative average traces of T-current activation before and after perfusion with TTA-P2 are shown in Figure 4*F*, orange trace, Cs with ATP; 11 cells, eight animals; violet trace, same cells with TTA-P2. Plots in Figure 4*G*,*H* show that TTA-P2 reduced current density by 84% and decreased the average maximal peak T-current amplitude from 573 ± 71 to 89 ± 24 pA.

### Pharmacological inhibition of T-currents with TTA-P2 reduces tonic and rebound burst firing

**Figure 5.**
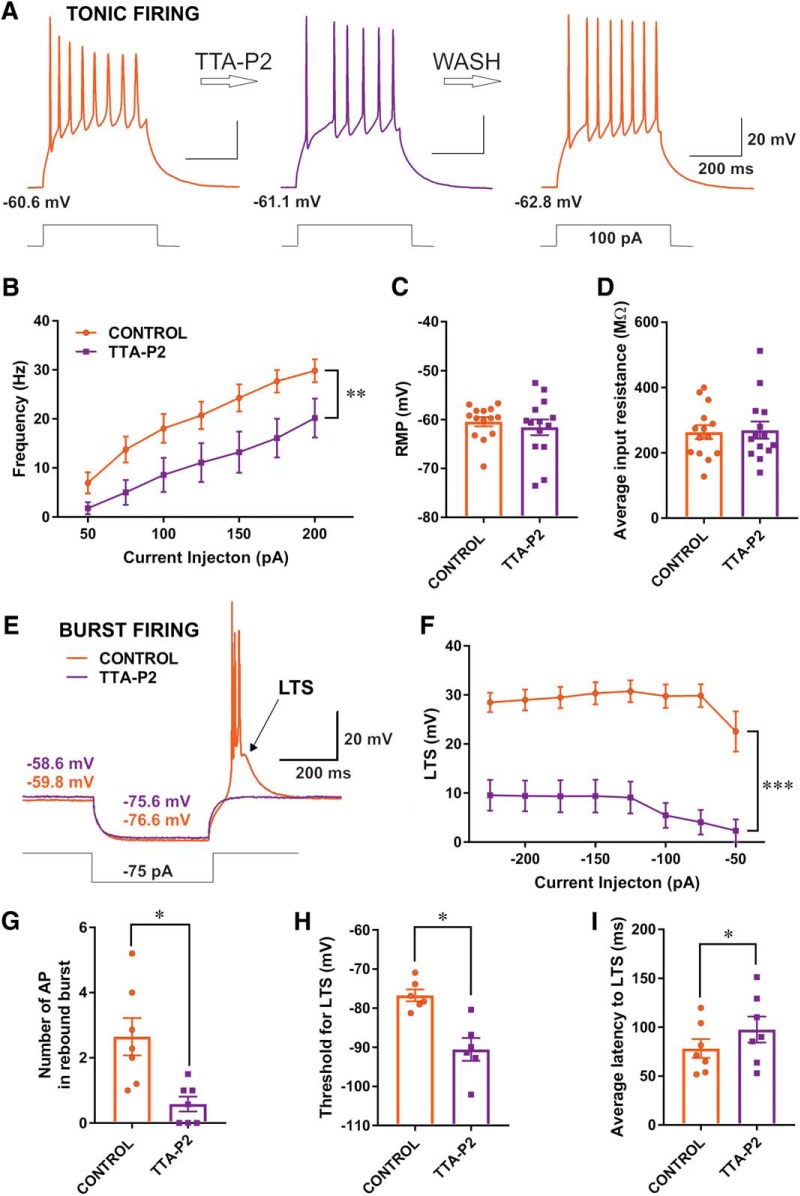
TTA-P2 reduced tonic and rebound burst firing in CeM neurons. ***A***, Original traces from a representative neuron in the CeM before application of TTA-P2 (left panel, orange trace), after application of TTA-P2 (middle panel, violet trace) and after wash (right panel, orange trace). Resting membrane potentials (shown in the lower left corner of each panel) show active membrane responses to a depolarizing (100 pA) current injection. ***B***, TTA-P2 reduced tonic AP firing frequency across all current pulses (from 50 to 200 pA); two-way RM ANOVA (both factors): interaction (*F*_(6,78)_ = 1.02, *p* = 0.418), current injection (*F*_(6,78)_ = 39.61, *p* < 0.001), and effect of TTA-P2 (*F*_(1,13)_ = 10.91, *p* = 0.006). ***C***, TTA-P2 had very little effect on average resting membrane potential (paired two-tailed *t* test, *t*_(13)_ = 0.91, *p* = 0.380). ***D***, TTA-P2 also had little effect on average input resistance of CeM neurons (paired two-tailed *t* test, *t*_(13)_ = 0.39, *p* = 0.702). ***E***, Original traces from a representative CeM neuron showing postinhibitory rebound burst-firing before (orange trace) and after application of TTA-P2 (violet trace). Burst-firing was induced by injection of a hyperpolarizing (–75 pA) current during 400 ms. Note that TTA-P2 completely abolished active membrane response to the current injection. ***F***, Graph of averaged traces of CeM neurons shows LTS amplitude of LTSs was almost completely abolished by the application of TTA-P2 across all hyperpolarizing current pulses from –50 to –225 pA [two-way RM ANOVA (both factors): interaction (*F*_(7,84)_ = 1.71, *p* = 0.116), current injection (*F*_(7,84)_ = 4.32, *p* < 0.001), and effect of TTA-P2 (*F*_(1,12)_ = 51.03, *p* < 0.001)]. ***G***, Bar graph showing TTA-P2 significantly reduced the number of APs in rebound burst (paired two-tailed *t* test, *t*_(6)_ = 2.94, *p* = 0.026). ***H***, Bar graph showing TTA-P2 significantly increased the threshold for the occurrence of LTS (paired two-tailed *t* test, *t*_(5)_ = 3.44, *p* = 0.018). ***I***, Graph showing the average latency to LTS increased significantly with TTA-P2 (paired two-tailed *t test*, *t*_(6)_ = 2.46, *p* = 0.049); *p < 0.05, **p < 0.01, ***p < 0.001.

It has been reported that CeM neurons may fire APs in both tonic and postinhibitory rebound burst firing modes (Jhangiani-Jashanmal et al., 2016). Furthermore, it has been well established that T-channels in other thalamic nuclei are crucial contributors to burst firing mode, since during hyperpolarization more T-channels recover from inactivation and may be readily activated during return to resting membrane potentials. At more depolarized membrane potentials, when most of the T-channels are inactivated, the tonic firing mode is the predominant form of spike discharge. to examine consequences of T-current inhibition with TTA-P2 on intrinsic excitability of CeM neurons, we monitored their firing patterns of APs using current-clamp recordings. In these experiments, we injected a series of depolarizing and hyperpolarizing currents of the same duration to assess tonic firing, membrane input resistance, and to quantify rebound burst firing. Perfusion with TTA-P2 reversibly inhibited the number of APs evoked by depolarizing current injections of 100 pA (Fig. 5*A*). When CeM neurons were perfused with TTA-P2 and tonic firing frequency was recorded at –60 mV across all current pulses from 50 to 200 pA, it resulted in a significant decrease of ∼50% when compared with control conditions (Fig. 5*B*; 14 cells, 10 animals). In contrast, TTA-P2 had very little effect on average resting membrane potential (Fig. 5*C*; two-tailed paired *t* test, *t*_(13)_ = 0.91, *p* = 0.380) and average input resistance (Fig. 5*D*; two-tailed paired *t* test, *t*_(13)_ = 0.39, *p* = 0.702).

CeM cells exhibit a characteristic burst-firing mode after periods of membrane hyperpolarization, which is typical of many thalamic neurons. Traces from a representative neuron with a hyperpolarizing current injection of 75 pA before application of TTA-P2 (orange trace) and after application of TTA-P2 (violet trace) are shown in Figure 5*E*. When injection of hyperpolarizing current was sufficient to remove inactivation of T-currents, neurons showed rebound LTSs and burst-firing mode indicated by a barrage of APs that crown LTSs in the control conditions. Furthermore, the violet trace in Figure 5*E* shows that TTA-P2 completely abolished LTS and rebound burst-firing in this neuron. The graph showing the average inhibitory effect of TTA-P2 on the amplitude of baseline LTS from multiple CeM neurons (*n* = 14) over the range of escalating current injections is presented in Figure 5*F*. Furthermore, we found a decrease of ∼2.5-fold in the average number of APs during LTS after application of TTA-P2 (Fig. 5*G*). Additionally, the neuronal cell membrane needed to be significantly more hyperpolarized to reach the threshold for LTS after TTA-P2 application (Fig. 5*H*). Finally, we found that TTA-P2 application also increased the average latency to LTS of ∼20% (Fig. 5*I*). Overall, our data indicate that selective inhibition of T-currents in CeM neurons with TTA-P2 significantly constrains both tonic and burst-firing modes as determined by several excitability measures.

### Cytosolic ATP regulates neuronal excitability

**Figure 6.**
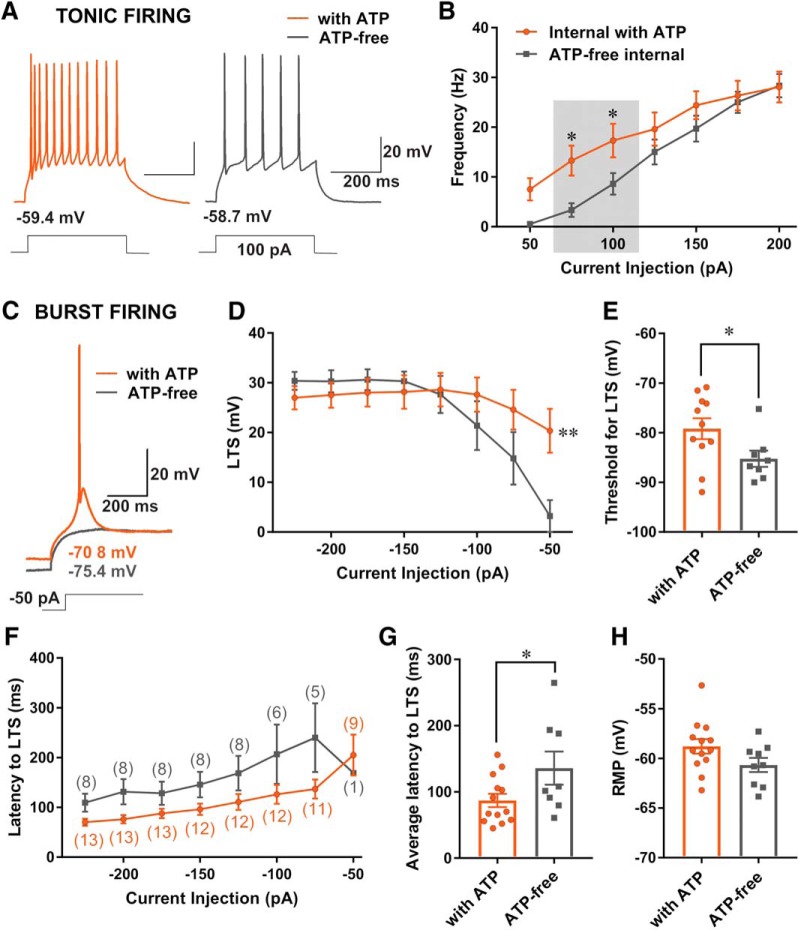
Cytosolic ATP regulates neuronal excitability. ***A***, Original traces from representative neurons in the CeM recorded with ATP (left panel, orange trace) and ATP-free internal solutions (right panel, gray trace) show active membrane responses (lower left corner of panels) to a depolarizing (100 pA) current injection. ***B***, Graph of averages of tonic AP firing frequency and current injections of 50–200 pA from multiple experiments shows that ATP-free internal solution significantly reduced firing frequency during smaller depolarizing current injections [two-way RM ANOVA: interaction (*F*_(6120)_ = 3.18, *p* = 0.006), current injection (*F*_(6120)_ = 74.67, *p* < 0.001), and ATP (*F*_(1,20)_ = 2.13, *p* = 0.159; shaded area indicates uncorrected Fisher’s LSD test presented on graph)]. ***C***, Representative traces of the CeM neurons show active membrane responses to hyperpolarizing (–50 pA) current injections in conditions with an ATP internal solution (orange trace) and with ATP-free internal solution (gray trace). ***D***, Graph of averages of LTS amplitudes from multiple experiments following escalating current injections ranging from –50 to –225 mV. Recordings with ATP-free internal solution (gray line) show a significant decrease in LTS amplitude as a response to –50-pA hyperpolarizing current injection [two-way RM ANOVA: interaction (*F*_(7133)_ = 5.57, *p* < 0.001), current injection (*F*_(7133)_ = 15.76, *p* < 0.001), and ATP (*F*_(1,19)_ = 0.50, *p* = 0.488, Sidak’s *post hoc* presented on figure)]. ***E***, Bar graph shows addition of ATP in the internal solution lowers the threshold for LTS occurrence [unpaired two-tailed *t* test, *t*_(17)_ = 2.12, *p* = 0.048]. ***F***, Graph showing latency to LTS (expressed in ms) in conditions with ATP (orange color) and without ATP (gray color) after escalating hyperpolarizing current injections; numbers in parentheses indicate respective number of cells in each group. ***G***, Bar graph showing exclusion of ATP from the internal solution during hyperpolarizing current injection (from –125 to –225 pA) significantly extended time for LTS formation by increasing the cumulative average latency to LTS (unpaired two-tailed *t* test, *t*_(19)_ = 2.10, *p* = 0.049). ***H***, Bar graph indicates very little difference between the average resting membrane potential recorded with and without ATP (unpaired two-tailed *t* test, *t*_(20)_ = 1.78, *p* = 0.090); *p < 0.05, **p < 0.01.

Previous studies demonstrated that high-frequency burst-firing pattern associated with thalamic LTS closely depends on T-current kinetics and that even minimal modifications in biophysical properties of the T-current can condition neuronal responses to synaptic potentials and profoundly affect neuronal excitability (Tscherter et al., 2011). Hence, we hypothesized that ATP modulation of biophysical properties of T-currents, such as voltage-dependent inactivation and slowing of recovery from inactivation, as well as decreased T-current density could, in turn, diminish excitability of CeM neurons. First, we examined the effect of the presence of ATP in the internal solution on the tonic firing mode of CeM neurons. CeM neurons exposed to a depolarizing current injection of 100 pA with an ATP-free internal solution fired less APs when compared to neurons with ATP with same depolarizing current injection (Fig. 6*A*). In addition, Figure 6*B* shows an ATP-free internal solution with current injections of 50–200 pA reduced the average firing frequency of tonic APs recorded from resting membrane potential (9 cells) when compared to recordings with ATP in the internal solution (13 cells). Although exclusion of ATP from the internal solution did not have a statistically significant effect on overall firing rate across all current injection steps (50–200 pA in 25-pA increments), recorded firing frequency with smaller current injections (75 and 100 pA) showed a statistically significant reduction in depolarization-induced firing of ∼75% and 50%, respectively (Fig. 6*B*, shaded area).

Next, we examined the effects of an ATP-free internal solution on the burst-firing mode of CeM neurons. Representative traces after hyperpolarizing current injection of 50 pA with internal solution containing ATP (orange trace) and ATP-free internal (gray trace) are presented in Figure 6*C*. A current injection of 50 pA with an ATP-free internal solution failed to evoke a LTS, in contrast to the prominent LTS evoked with an internal solution containing ATP. The average effect of ATP in internal solution on LTS amplitude is illustrated in the graph shown in Figure 6*D*. Recordings of burst-firng of CeM neurons during escalating current injections from –50 to 225 mV showed a significant decrease in LTS amplitude as a response to –50-pA hyperpolarizing current injection in the absence of ATP compared with recordings conducted with ATP in the internal solution (Fig. 6*D*, ATP-free, gray line, with ATP, orange line). In contrast to TTA-P2, which inhibited the LTS amplitude across all currents (Fig. 5*F*), a statistically significant decrease in LTS with an ATP-free internal solution occurred only at the initial hyperpolarizing current injection of 50 pA (Fig. 6*D*, Sidak’s *post hoc*). However, similar to the pharmacological inhibition of the channel seen with TTA-P2 (Fig. 5*H*), a greater hyperpolarization of the neuronal cell membrane potential was required to reach the threshold for LTS with an ATP-free solution (Fig. 6*E*, gray bar, eight cells) than in the recordings with ATP (Fig. 6*E*, orange bar, 11 cells). We also measured the latency to LTS across all hyperpolarizing current injections ranging from 50–225 pA in 25-pA incremental steps (Fig. 6*F*). Bar graphs (Fig. 6*G*) demonstrate the average cumulative latency to LTS was significantly different between the two groups as evidenced by longer latency of ∼45% in the group without ATP (–136.00 ± 25.13 ms, eight cells) when compared to the recordings done in the presence of ATP (–87.17 ± 9.98 ms, 13 cells). When we compared the average number of APs during LTS, we found no difference between the two internal solutions (data not shown). Similar to results obtained with TTA-P2, there was very little difference in the resting membrane potential between an internal solution with or without ATP (Fig. 6*H*; with ATP, –58.79 ± 0.72 mV, 13 cells; ATP-free, –60.67 ± 0.71 mV, nine cells). We also found the average input resistance of CeM neurons was not significantly different between the two groups (data not shown). Hence, increased excitability of CeM neurons recorded with an internal solution containing ATP cannot be attributed to alterations of passive membrane properties.

**Figure 7.**
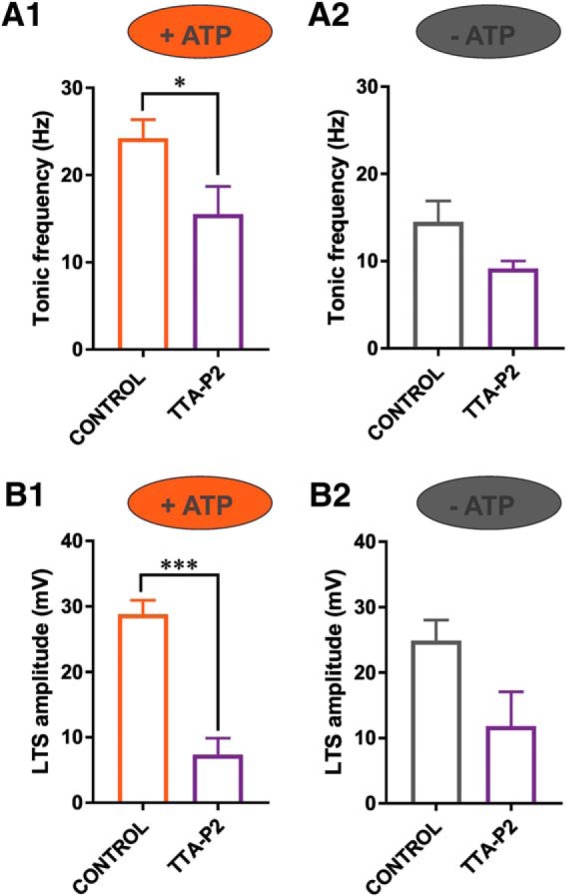
TTA-P2-induced inhibition of tonic and rebound burst-firing in CeM neurons is diminished in recordings with ATP-free internal solution. ***A1***, Bar graph showing TTA-P2 significantly reduced tonic firing frequency of APs with an internal solution containing ATP (paired two-tailed *t* test; *t*_(13)_ = 2.96, *p* = 0.011, *n* = 14). ***A2***, Bar graph showing TTA-P2 had a smaller effect on tonic firing frequency in recording conditions with an ATP-free internal solution (paired two-tailed *t* test; *t*_(5)_ = 14, *p* = 0.085, *n* = 6). ***B1***, Bar graph showing TTA-P2 significantly reduced LTS amplitude of CeM neurons in conditions with ATP in the internal solution (paired two-tailed *t* test; *t*_(12)_ = 7.14, *p* < 0.001, *n* = 13). ***B2***, Bar graph showing TTA-P2 had no significant effect on LTS amplitude in recording conditions with an ATP-free internal solution (paired two-tailed *t* test; *t*_(4)_ = 2.53, *p* = 0.065, *n* = 5); *p < 0.05, ***p < 0.001.

Finally, we explored whether there was an interaction between TTA-P2-induced inhibition of tonic and rebound burst-firing in the CeM and ATP-induced decrease in excitability (Fig. 7). We found TTA-P2 had a less prominent effect on the reduction of tonic and burst firing frequency in an ATP-free internal solution compared with an ATP-rich internal solution (Fig. 7, compare *A1*, *A2*, and *B1*, *B2*). As expected, TTA-P2 perfusion recorded with an ATP-containing internal solution resulted in a statistically significant reduction in the average cumulative tonic firing frequency of ∼30% (Fig. 7*A1*; 14 cells, paired two-tailed *t* test; *t*_(13)_ = 2.96, *p* = 0.011) and average reduction in cumulative LTS amplitude of ∼75% (Fig. 7*B1*; 13 cells, paired two-tailed *t* test; *t*_(12)_ = 7.14, *p* < 0.001). In contrast, the inhibitory effect of TTA-P2 perfusion with an ATP-free internal solution on average LTS amplitude (Fig. 7*A1*; five cells) and tonic firing frequency (Fig. 7*B1*; six cells) was not significant (*p* > 0.05). These data suggest that TTA-P2 and ATP-free solutions may diminish neuronal excitability in the CeM by the similar mechanisms that involve T-channels.

## Discussion

Our findings demonstrate that T-channels play an important role in the regulation of excitability of rat CeM neurons. Importantly, the selective pharmacological blocker, TTA-P2, indicates T-channels play a major role in shaping the output of CeM neurons by regulation of both depolarization-induced tonic firing mode and hyperpolarization-induced rebound burst-firing mode. Furthermore, we found that T-channel activity in CeM neurons is strongly regulated by cytosolic ATP, which modulates several key aspects of its function, including current density, recovery form inactivation, and voltage-dependent inactivation. Consequently, use of an ATP-free internal solution decreased excitability of CeM neurons.

### CeM neurons have prominent T-currents that contribute to both tonic and burst-firing modes

Our voltage-clamp experiments revealed similar biophysical properties and large current densities of T-channels in rat CeM neurons that correspond well with studies investigating T-currents in other thalamocortical neurons such as the VB nucleus (Eckle and Todorovic, 2010; Eckle et al., 2012) and the adjacent rostral nucleus reuniens (Walsh et al., 2017). Using dynamic-clamp experiments, Tscherter et al. (2011) showed that even minimal differences in biophysical properties of T-channels could shape the precise ﬁring patterns of thalamic neurons in the VB nucleus. For example, the alteration of the control slope factor of channel inactivation remarkably changed the excitability of these neurons. Furhermore, the maximal excitability was observed for the slope factor of *k* = 6, which corresponds very well with the values obtained in our experiments with CeM neurons.

Pharmacological inhibition of T-channels with TTA-P2 in our experiments completely abolished amplitudes of LTSs and subsequent burst-firing of CeM neurons. This was expected, based on other studies in CNS neurons that express abundant T-currents. However, we also found that TTA-P2 partially inhibited depolarization-induced tonic firing as well. This finding may be somewhat surprising since T-channels are largely inactivated at the more depolarized potentials. Nonetheless, Swensen and Bean (2003) found that T-currents are responsible for most of the total calcium current between the spikes in Purkinje neurons. Our finding is also in agreement with a study by Deleuze et al. (2012), who showed that the activation of T-channels plays a crucial role in tonic firing of thalamocortical neurons in the VB nucleus, and thus may regulate the transfer of sensory inputs during wakefulness. Toward this end, we recently reported that the Ca_V_3.1 isoform of T-channels significantly contributes to both burst and tonic firing modes of subicular neurons (Joksimovic et al., 2017).

### Functional significance of ATP regulation of T-channel-dependent excitability of CeM neurons

One of the important findings of this study is that cytosolic ATP may be an endogenous modulator of T-channel function in CeM neurons. Specifically, when cells were dialyzed with ATP-free solution, T-currents had decreased current densities, availability of T-channels was greatly diminished at physiologic membrane potentials due to enhanced inactivation, and recovery of T-channels from inactivation was significantly slower. We then demonstrated in subsequent current-clamp recordings that an ATP-free solution had decreased excitability of CeM neurons, which exhibited slower frequency of firing of tonic APs, decreased amplitudes of LTS, increased threshold, and longer latency for LTS generation.

During wakefulness and paradoxical, rapid eye movement (REM) sleep episodes most thalamic neurons are likely to be depolarized, and cells will be in tonic firing mode. In contrast, during slow-wave, non-REM (NREM) or delta sleep and during anesthesia, thalamic neurons will be hyperpolarized, which will allow T-type channels to be deinactivated and burst firing mode will be more prominent. Hence, given the known role of the CeM in awareness and consciousness, we propose that cytosolic ATP could be an endogenous regulatory mechanism by which T-channels can functionally gate sensory transmission and arousal *in vivo*. Furthermore, cytosolic ATP and T-type channel-dependent regulation of neuronal excitability may provide an important link between energy metabolism and neuronal firing, which could mediate homeostatic responses to neuronal hyper-excitability that occurs in the thalamus in disorders such as epilepsy, tinnitus, neurogenic pain syndromes, and possibly other certain psychiatric diseases collectively termed “thalamocortical dysrhythmias” (for reviewe, see Llinás et al., 2005). Furthermore, both human and animal studies have indicated that the thalamus is deactivated during general anesthesia (Angel, 1991; Alkire et al., 2000).

Moreover, the significant positive correlation was observed between the surge in ATP and NREM delta activity recorded in EEG during spontaneous sleep (Dworak et al., 2010). In rodents, there is an increase in delta power in EEG during NREM sleep and the key role of T-channels in delta sleep is well established (for review, see Crunelli et al., 2014). However, the mechanisms of T-channels activation during NREM sleep are not well studied. We speculate that the surge in ATP could be one of the mechanisms that potentiate T-channels in CeM and consequently promotes transitions into NREM sleep from awake states. Hence, this energy-dependent regulatory mechanism of T-channels and thalamic sensory transmission may underlie changes in sensory transmission that occur during the sleep-wake cycle as well as transitions between awake and anesthetized states as energy is depleted and replenished.

### Mechanisms of modulation of T-type cannels and CeM neuron excitability and by ATP

The cytosolic ATP is generated from ADP, the main regulator of ATP synthesis, during glycolysis, citric acid cycle and by oxidative phosphorylation in the mitochondria (Sperlágh and Vizi, 1996). Although cells store ATP in all types of synaptic vesicles, ATP can be found in the cytoplasm in millimolar range (Sperlágh and Vizi, 1996). Hence, it is interesting to speculate on possible mechanisms of ATP-mediated modulation of T-channels in CeM neurons. One possibility is that use of ATP- and GTP-containing salts in the internal solution might have nonspecific effects, causing electrostatic interactions with voltage sensors and surface potential, as described for cytosolic divalent cations and ATP-sensitive potassium channels in cardiac myocytes (Deutsch et al., 1994). We believe this possibility is unlikely because one would expect that changes in surface potential or any other nonspecific effect would equally affect both voltage-dependent activation and voltage-dependent inactivation parameters of T-channels. In contrast, we found that use of ATP-free internal solutions selectively affected properties of voltage-dependent inactivation, which was shifted to the more hyperpolarized potentials, while properties of voltage-dependent activation were spared in the identical recording conditions.

Interestingly, in the VB nucleus T-type currents recorded with ATP in the internal solution showed an increase both in the current amplitude and macroscopic inactivation kinetics, while dialysis of the neuron with an ATP-free solution suppresses the T-current potentiation (Leresche et al., 2004). It has been shown that cytosolic ATP-induced modulation of T-currents in the VB and other sensory thalamic nuclei (but not in nonspecific midline thalamic nuclei) expressing Ca_V_3.1 currents occurs when the channels are inactivated and is slowly removed when they recover from inactivation and remain in closed states (Leresche et al., 2004). Interestingly, although T-channel potentiation by ATP in VB neurons does not affect LTS amplitude and duration, the latency of the LTS onset decreases in the absence of ATP (Bessaïh et al., 2008). Our findings in CeM neurons appear very different from those in VB neurons; we found that an ATP-free solution did not affect macroscopic current inactivation kinetics and had a stronger inhibitory effect on recovery from inactivation at –120 than –90 mV. Furthermore, decreased T-current densities over the wide range of membrane potentials in ATP-free conditions strongly suggests that ATP interacts with both closed and inactive states of the channels. However, regardless of different biophysical mechanisms of T-channel modulation by cytosolic ATP in CeM and VB thalamic neurons, in both regions ATP potentiates T-channel function and supports neuronal excitability.

It has been shown that a multitude of endogenous modulators can differentially regulate all three isoforms of T-type channels (Zhang et al., 2013). As voltage-gated channels, T-type channels are primarily regulated by alterations in membrane potential, but different hormones and neurotransmitters can regulate its activity by activating G proteins or protein kinases (Chemin et al., 2006; Iftinca and Zamponi, 2009). Earlier molecular studies have established that the dominant isoform of T-type channels in the CeM and VB nucleus is Ca_V_3.1 (Talley et al., 1999). The specific molecular mechanisms of T-current modulation by cytosolic ATP in CeM and VB thalamic neurons are not currently known. One possibility may involve phosphorylation reactions, which was suggested by previous studies on VB neurons (Leresche et al., 2004). However, although there is a good evidence that phosphorylation can regulate Ca_V_3.2 channels (Iftinca et al., 2007; Blesneac et al., 2015), the influence of phosphorylation reactions on Ca_V_3.1 channels has not been well studied. Some studies revealed potentiation of recombinant Ca_V_3.1 channels by protein kinase C, temperature-dependent phosphorylation, and inhibition by Rho-associated kinase (Iftinca et al., 2006; Park et al., 2006; Chemin et al., 2007; Iftinca et al., 2007). However, similar findings are not available for native brain tissues. Further studies are needed to decipher the molecular mechanisms of cytosolic ATP modulation of T-currents and neuronal excitability in the CeM and VB neurons.

### Concluding remarks

The main goal of this study was to investigate and characterize properties of T-type calcium channels in the CeM thalamic nucleus, a brain structure that is critically involved in the control of consciousness. We found that T-type channels are essential in the genesis of high-frequency rebound bursts following membrane hyperpolarization, such as those that can occur during IPSPs. In addition, since thalamic T-channels are involved in consolidating EPSPs at depolarized potentials (Deleuze et al., 2012), we examined the influence of T-type channels on tonic firing properties of CeM neurons as well. We found that selective pharmacological inhibition of T-channels and reduction of T-channel activity by exclusion of ATP in the internal solution constrained both tonic and burst firing modes of CeM neurons. We conclude that T-channels are critical regulators of CeM thalamo-cortical circuit output. Furthermore, we demonstrated that cytosolic ATP can fine tune T-channel dependent excitability of CeM neurons. Hence, we propose that cytosolic ATP could be an endogenous regulatory mechanism in which T-channels may functionally gate sensory transmission and arousal *in vivo*.

## Synthesis

Reviewing Editor: Vivian Budnik, University of Massachusetts Decisions are customarily a result of the Reviewing Editor and the peer reviewers coming together and discussing their recommendations until a consensus is reached. When revisions are invited, a fact-based synthesis statement explaining their decision and outlining what is needed to prepare a revision will be listed below. The following reviewer(s) agreed to reveal their identity: Philippe Lory, John Huguenard.

The authors have now responded satisfactory to reviewer's comment and besides the minor comment below, the manuscript is now ready for publication.

Page 12 : « ... the time constants presented in Figures 3B and 3C ... » (not 4B and 4C)
